# Secreted Acid Phosphatase (SapM) of *Mycobacterium tuberculosis* Is Indispensable for Arresting Phagosomal Maturation and Growth of the Pathogen in Guinea Pig Tissues

**DOI:** 10.1371/journal.pone.0070514

**Published:** 2013-07-26

**Authors:** Rupangi Verma Puri, P. Vineel Reddy, Anil K. Tyagi

**Affiliations:** Department of Biochemistry, University of Delhi South Campus, New Delhi, India; Institut de Pharmacologie et de Biologie Structurale, France

## Abstract

Tuberculosis (TB) is responsible for nearly 1.4 million deaths globally every year and continues to remain a serious threat to human health. The problem is further complicated by the growing incidence of multidrug-resistant TB (MDR-TB) and extensively drug-resistant TB (XDR-TB), emphasizing the need for the development of new drugs against this disease. Phagosomal maturation arrest is an important strategy employed by *Mycobacterium tuberculosis* to evade the host immune system. Secretory acid phosphatase (SapM) of *M.tuberculosis* is known to dephosphorylate phosphotidylinositol 3-phosphate (PI3P) present on phagosomes. However, there have been divergent reports on the involvement of SapM in phagosomal maturation arrest in mycobacteria. This study was aimed at reascertaining the involvement of SapM in phagosomal maturation arrest in *M.tuberculosis*. Further, for the first time, we have also studied whether SapM is essential for the pathogenesis of *M.tuberculosis*. By deleting the *sapM* gene of *M.tuberculosis*, we demonstrate that MtbΔ*sapM* is defective in the arrest of phagosomal maturation as well as for growth in human THP-1 macrophages. We further show that MtbΔ*sapM* is severely attenuated for growth in the lungs and spleen of guinea pigs and has a significantly reduced ability to cause pathological damage in the host when compared with the parental strain. Also, the guinea pigs infected with MtbΔ*sapM* exhibited a significantly enhanced survival when compared with *M.tuberculosis* infected animals. The importance of SapM in phagosomal maturation arrest as well as in the pathogenesis of *M.tuberculosis* establishes it as an attractive target for the development of new therapeutic molecules against tuberculosis.

## Introduction

Inspite of a rapid advancement in our understanding of the biology of *Mycobacterium tuberculosis*, the success towards the control of tuberculosis (TB) has been less than satisfactory as is evident from ∼9 million new cases of the disease that still occur globally every year [1]. The emergence of multidrug-resistant (MDR) and extensively drug-resistant (XDR) strains of the pathogen and the rising number of HIV-TB co-infections have made the situation even more precarious. There are an estimated 440,000 new cases of MDR-TB, 25,000 cases of XDR-TB and 1.1 million cases of TB-HIV co-infection globally every year [1], [2]. Despite the multitude of immune defense mechanisms that the host deploys against *M.tuberculosis*, the pathogen can continue to persist owing to its subtle tactics [3]. *M.tuberculosis* is inhaled via the droplet nuclei and then taken up by alveolar macrophages [3]. The outcome of the infection largely depends on the interaction between the host and the pathogen, especially within the macrophages [4]. There are several ways in which *M.tuberculosis* modulates the macrophage defenses to promote its own survival and the inhibition of phagosomal maturation is one of the best characterized mechanisms [4], [5], [6], [7].

Phagolysosomal fusion requires the presence of phosphotidylinositol 3-phosphate (PI3P) on the phagosomes [8]. This lipid component is involved in the docking of rab effector proteins such as early endosomal autoantigen 1 (EEA1) and hepatocyte growth factor-regulated tyrosine kinase substrate (Hrs) that are important for phagosomal maturation [8], [9], [10]. SapM is a secretory phosphatase that has been demonstrated to dephosphorylate PI3P [11], [12]. However, several studies have presented divergent observations regarding the involvement of SapM in arresting phagosomal maturation in mycobacteria [11], [13], [14]. Based on a study with BCG, it has been reported that phagosomes that harbour killed BCG persistently carry PI3P, however, PI3P is removed from the phagosomes that harbour live BCG [11]. An *in vitro* fusion assay in the presence of purified SapM protein from *M.tuberculosis*, which has been demonstrated to hydrolyse PI3P, further suggested that SapM inhibits phagosome-late endosome fusion *in vitro* [11]. Based on these observations SapM has been implicated in phagosome maturation arrest in *M.tuberculosis* [11]. However, contradictory observations on the role of SapM in phagosomal maturation arrest have also emanated from the colocalization experiments which employed Mf4/4 murine macrophages infected with either BCG or its *sapM* mutant and led to the observation that SapM is not necessary for the arrest of phagosome maturation in BCG [14]. Further, it has been reported that BCG and its *sapM* mutant exhibit no significant difference in the survival and replication in macrophages [14]. However, based on the phagosomal maturation studies involving the infection of THP-1 macrophages with wild-type *M.tuberculosis* and its *fbpA* mutant (Δ*fbpA*), *sapM* mutant (Δ*sapM*) as well as double knock out mutant (Δ*sapM*Δ*fbpA*), it has been reported that the double knock out strain was extensively colocalized with the lysosomal markers followed by Δ*sapM,* Δ*fbpA* and the parental strain, indicating thereby the involvement of SapM in the phagosomal maturation arrest [13]. Thus, the role of SapM in arresting the phagosomal maturation still remains a question that has not been answered beyond doubt. More importantly, there has been no study to evaluate the role of SapM at genetic level by employing the *sapM* mutant of *M.tuberculosis* and conducting animal experiments to show the role of SapM in the pathogenesis of *M.tuberculosis*. To demonstrate the function of a gene in the phenomena such as phagosomal maturation and its consequential influence on the host-pathogen interaction and pathogenesis of *M.tuberculosis*, it would be desirable that the *in vitro* studies be supported by animal experiments in a relevant model and Koch’s postulates be satisfied.

In this study, we have employed a *sapM* mutant of *M.tuberculosis* along with the parental strain to reascertain the involvement of SapM in phagosomal maturation arrest. Further, we have evaluated the influence of *sapM* mutation on the growth and pathogenesis of *M.tuberculosis* in guinea pigs.

## Materials and Methods

### Bacterial Strains and Growth Conditions

*Escherichia coli* strains XL-1 Blue (Stratagene, Heidelberg, Germany) and HB101 (Life Technologies, CA, USA) were used for cloning and were grown in Luria-Berteni (LB) broth or on LB agar. Mycobacterial strains were grown on Middlebrook (MB) 7H11 agar supplemented with 10% OADC (oleic-acid albumin dextrose catalase) and 0.2% glycerol or in MB7H9 broth supplemented with 10% ADC (albumin dextrose catalase), 0.2% glycerol and 0.05% Tween 80 at 37°C with shaking at 200 rpm. For the generation of mutants, *M.tuberculosis* Erdman transformed with pJV53 and thereby overexpressing the recombineering proteins that enhance the frequency of genetic recombination was employed, as described previously [15]. Thus, for all the experiments carried out in this study, we have used *M.tuberculosis* Erdman/pJV53 as the parental strain. Kanamycin and chloramphenicol were used at concentrations of 25 µg/ml and 30 µg/ml, respectively. Hygromycin was used at a concentration of 50 µg/ml for mycobacteria or at 150 µg/ml for *E.coli*.

### Disruption of *sapM* in *M.tuberculosis* and Genetic Complementation of the Mutant

Primers SapM-F1 (5′ aatattggggtaccaccatcgggtcaagcacc 3′) and SapM-R1 (5′ ttaatatctagaatgatggcggcctgcgagc 3′) were designed to amplify Amplicon I (699 bp), comprising of 200 bp 5′ proximal region of *sapM* and 499 bp sequence immediately upstream to *sapM*, while the primers SapM-F2 (5′ aatattccgctcgagcttgtgacctgggacgaag 3′) and SapM-R2 (5′ ttgatcgactagtctcgaattccagcgtttattg 3′) were used for the amplification of Amplicon II (701 bp), comprising of 200 bp 3′ distal region of *sapM* and 501 bp sequence immediately downstream to *sapM*. The amplicons I and II were PCR amplified and cloned into the vector pYUB854 flanking the hygromycin resistance cassette at *Kpn*I/*Xba*I and *Xho*I/*Spe*I restriction sites, respectively, to generate pYUBΔ*sapM* [16]. A 3.4 kb fragment (Δ*sapM::hyg*), was excised from pYUBΔ*sapM* by employing *Kpn*I/*Spe*I and the resulting linear Allelic Exchange Substrate (AES) was electroporated into *M.tuberculosis* as described earlier to generate the *sapM* mutant of *M.tuberculosis* (MtbΔ*sapM*) [15].

To facilitate genetic complementation of the MtbΔ*sapM*, primers SapM-F3 (5′ gatatcggatcccagcgctgcccggaagccggcgttgtc 3′) and SapM-R (5′ gatatcggatccctagtcgccccaaatatcggttattg 3′) were designed to amplify the *sapM* gene (900 bp) along with 200 bp region upstream of the SapM translational start site, containing the *sapM* promoter. This region (1.1 kb) was PCR amplified and cloned into the *Bam*HI site of the mycobacterial shuttle vector, pSD7 [17]. The resulting plasmid pSD7.SapM, was introduced into MtbΔ*sapM* mutant by electroporation to generate the complemented strain, MtbΔ*sapM*Comp. For the confirmation of disruption of *sapM* by PCR amplification, primers SapM-F (5′ tcggcgctgtcccacaagtg 3′) and SapM-R were employed.

### Determination of Phosphatase Activity

Acid phosphatase activity of SapM was determined by using an end point assay based on the detection of *p*-nitrophenol formed by using the substrate *p*-nitrophenyl phosphate (pNPP) (Sisco Research Laboratories Pvt. Ltd., Maharashtra, India) [12]. Briefly, 120 µg of mycobacterial culture filtrate (A_600 nm_ of 0.8), were separately incubated with 20 mM pNPP in 50 mM Tris-HCl buffer (pH 7.0) at 37°C for 20 min in dark (in a reaction volume of 500 µl). The absorbance was measured at 405 nm after addition of 500 µl of 1 M NaOH. Enzyme activity (U) was calculated by using the formula U (nmol/min/µg) = [E.V_total_]/[t.ε.d.µg of Culture filtrate] [where E is the mean (OD) of sample minus mean (OD) of blank (at 405 nm), V_total_ is the total sample volume (1000 µl), t is incubation time (20 min), ε is the extinction coefficient for pNPP (17.8 nM^−1^ cm^−1^) and d is the pathlength of light (1 cm)] [12].

### Infection of Human THP-1 Macrophages and Bacterial Enumeration

Human monocytic THP-1 cells were cultured in RPMI-GlutaMAX™ medium [containing 10% heat inactivated fetal bovine serum (FBS) and 1% antibiotic-antimycotic mix] (GIBCO Invitrogen Life Technologies, NY, USA) at 37°C in 5% CO_2_ and infected as described previously [18]. Briefly, THP-1 macrophages were seeded at 5×10^5^ cells per well in 24-well tissue culture plates and differentiated to macrophages by using 30 nM phorbol 12-myristate 13-acetate (PMA) (Sigma, MO, USA) for 16 h at 37°C in 5% CO_2_. Cells were washed with RPMI medium and rested for 2 h before infection in 1 ml RPMI medium supplemented with 10% FBS. The monolayers were infected with *M.tuberculosis* strains separately, at a multiplicity of infection of 1 bacterium per 3 THP-1 macrophages for 4 h at 37°C in triplicates, following which the monolayers were washed twice with the medium. Subsequently, extracellular bacteria were removed by treatment with 200 µg/ml amikacin for 2 h at 37°C. On days 0 (6 h), 2, 4 and 6, the infected macrophage monolayers (three wells per strain) were lysed with 1 ml of 0.025% SDS (Sigma, MO, USA) to release intracellular mycobacteria, which were then enumerated by plating serial dilutions on MB7H11 agar. Colonies were counted after 4 weeks of incubation at 37°C and the data was expressed as CFU/ml.

### Study of Phagosomal Maturation by using Confocal Microscopy

*M.tuberculosis* strains were labeled with Fluorescein isothiocyanate (FITC) (Sigma, MO, USA) as described previously with minor modifications [19], [20], [21]. Briefly, the *M.tuberculosis* cultures were grown to A_600 nm_ of 0.5. The culture was harvested, washed twice with 0.5 M sodium bicarbonate buffer (pH 9.5) and resuspended in the same buffer supplemented with 100 µg/ml FITC followed by an overnight incubation at 4°C. To evaluate the effect of FITC labeling on bacterial viability, *M.tuberculosis* cultures without the addition of FITC were subjected to similar treatments as described above and used as control. Thereafter, the bacteria were pelleted, washed twice with PBS (pH 7.4) and resuspended in RPMI-GlutaMAX™ medium containing 10% FBS. At the time of infection, the CFU was determined for FITC labeled and unlabelled *M.tuberculosis* cultures. Macrophages were seeded on poly-L-lysine (Sigma, MO, USA) coated glass coverslips within a 12-well plate at a density of 7.5×10^5^ macrophages per well, activated by addition of PMA and infected separately with 7.5×10^6 ^FITC labeled mycobacteria as described above. After a 4 h incubation, the THP-1 cells were washed twice with fresh RPMI media and treated with 200 µg/ml amikacin for 2 h at 37°C to remove extracellular bacteria. Subsequently, the THP-1 cells were incubated with 50 nM Lysotracker red DND-99 (Invitrogen Life Technologies, CA, USA) in RPMI (supplemented with 10% FBS) for 1 h. After this 7 h post infection period, the THP-1 cells were once washed with fresh RPMI media (supplemented with 10% FBS) and fixed with 4% paraformaldehyde in PBS. Coverslips were mounted by using ProLong® Gold antifade reagent (Invitrogen Life Technologies, CA, USA) and analysed by using Leica TCS SP5 confocal laser scanning microscope (Leica Microsystems, Mannheim, Germany). The fraction of FITC labelled mycobacteria that colocalized with LysoTracker was determined by analyzing ∼100 phagosomes (combined from at least five random fields) from three independent experiments. At the time of proceeding for the fixation of THP-1 cells infected with either *M.tuberculosis* or MtbΔ*sapM* or MtbΔ*sapM*Comp for evaluation under confocal microscope, the cells from duplicate wells in each case were lysed and plated on MB7H11 agar for bacterial enumeration.

### *In vivo* Guinea Pig Experiments

Pathogen-free out-bred female guinea pigs of the Duncan-Hartley strain (250 to 350 g) were obtained from the Disease Free Small Animal House Facility, Chaudhary Charan Singh Haryana Agricultural University, Hisar, India. The animals were maintained in a biosafety level 3 facility at University of Delhi South Campus. To study the influence of *sapM* mutation on the growth and pathogenesis of *M.tuberculosis*, guinea pigs were infected by the aerosol route with 10 to 30 bacilli of either *M.tuberculosis*, MtbΔ*sapM* or MtbΔ*sapM*Comp. Animals (n = 5) were euthanized at 4, 10 and 16 weeks post-infection by CO_2_ asphyxiation for bacterial enumeration in the lungs and spleen of infected animals. After dissecting the animals, the pathological investigations were carried out in the infected animals by: (i) visual scoring of gross lesions in the lung, liver and spleen and (ii) measurement of granulomatous inflammation in the histopathological sections of lung and liver.

The gross pathological damage was assessed by employing a scoring system as described previously [22]. Briefly, Mitchison’s virulence scoring system was modified and equal emphasis was given to every organ for visual scoring of lesions in lung, liver and spleen of guinea pigs infected with different strains of *M.tuberculosis* [22], [23]. Based on the extent of involvement of the organ, number and size of tubercles, areas of inflammation and damage due to necrosis, gross pathological scores were graded from 1–4 [22]. The organs displaying minimal, scanty, moderate or heavy involvement were scored as 1, 2, 3 or 4, respectively and represented graphically [22].

For histopathological evaluation, the right lung and a portion of left dorsal lobe of liver from the infected animals were removed and fixed in 10% buffered formalin. 5 µm thick sections from the formalin fixed, paraffin embedded tissues were stained with haematoxylin and eosin (H&E). The tissues were coded and the coded samples were evaluated under a light microscope by a certified pathologist having no knowledge of the experimental groups. Pulmonary and hepatic granuloma consolidations were graphically represented as % granuloma as described earlier [22].

To study the influence of disruption of *sapM* on the survival of infected animals, guinea pigs (n = 10) were infected as described above with either *M.tuberculosis*, MtbΔ*sapM* or MtbΔ*sapM*Comp. During the time period for which the survival was monitored, animals were regularly examined for change in body weight and general body condition as an indicator of disease progression.

### Ethics Statement

Protocols for all the animal experiments included in this manuscript along with the requirement of guinea pigs were reviewed and approved by the Institutional Animal Ethics Committee of University of Delhi South Campus, New Delhi, India (Ref. No. 1/IAEC/AKT/BIOCHEM/UDSC/14.10.2011). All animals were routinely cared for according to the guidelines of the CPCSEA (Committee for the Purpose of Control and Supervision of Experiments on Animals). The guinea pigs were euthanized by CO_2_ asphyxiation and all efforts were made to minimize animal suffering.

### Statistical Analyses

For the comparisons between the groups for the determination of (i) phosphatase activity in the mycobacterial culture filtrates, (ii) phagosomal maturation in the infected THP-1 cells and (iii) bacillary load in the lungs or spleen of infected guinea pigs, one-way analysis of variance (ANOVA) with the Tukey post test was employed. For the comparison of growth of mycobacterial strains in THP-1 cells, two-way ANOVA with the Bonferroni multiple comparison test was employed. For comparisons between the groups for the analyses of gross pathological and histopathological damage, the nonparametric Kruskal-Wallis test followed by the Mann-Whitney U test was employed. Survival of guinea pigs was analyzed by using the log-rank (Mantel-Cox) Test. Differences were considered significant when *P*<0.05. For the statistical analysis and generation of graphs, Prism 5 software (version 5.01; GraphPad Software Inc., CA) was used.

## Results

### Construction and Characterization of *sapM* Mutant of *M.tuberculosis*

**Figure 1:**
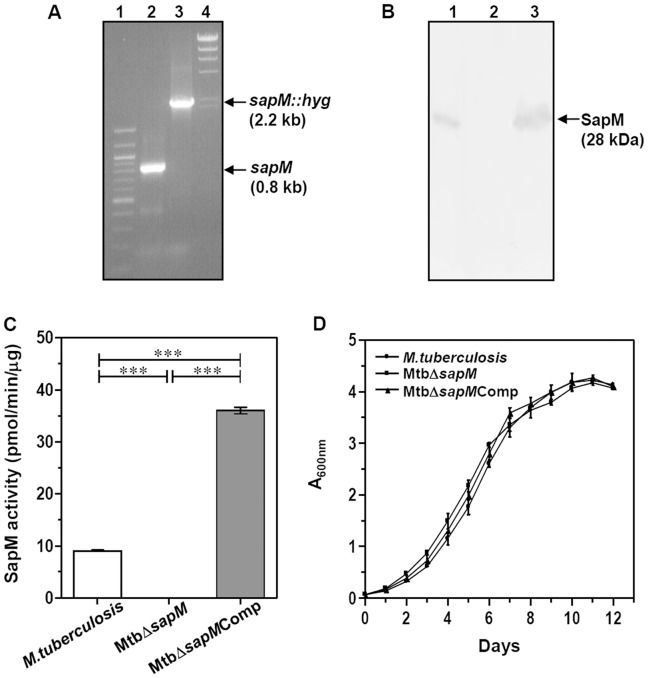
Characterization of MtbΔ*sapM* mutant. (A) Confirmation of *sapM* gene deletion in *M.tuberculosis* by PCR. PCR was carried out by employing primers SapM-F and SapM-R to obtain 0.8 kb amplification in *M.tuberculosis* (lane 2) and 2.2 kb amplification in MtbΔ*sapM* (lane 3). 100 bp and λ*Hind*III markers were loaded in lanes 1 and 4, respectively. (B) Confirmation of *sapM* deletion in *M.tuberculosis* and complementation of *sapM* gene in the mutant by immunoblot analysis. 10 µg of culture filtrate of *M.tuberculosis* (lane 1), MtbΔ*sapM* (lane 2*)* and MtbΔ*sapM*Comp (lane 3) were loaded on a 12% polyacrylamide gel and subjected to electrophoresis. Anti-SapM polyclonal antibody was employed to detect SapM that migrated as a protein band corresponding to a molecular mass of 28 kDa in the culture filtrate of *M.tuberculosis* (lane 1) and MtbΔ*sapM*Comp (lane 3) while the disruption of *sapM* in MtbΔ*sapM* (lane 2) was confirmed by the absence of protein expression. (C) Disruption of *sapM* results in the loss of phosphatase activity. 120 µg of culture filtrate from *M.tuberculosis*, MtbΔ*sapM* and MtbΔ*sapM*Comp were incubated with 20 mM pNPP and absorbance was measured at 405 nm as described in the materials and methods. Phosphatase activity was detected in *M.tuberculosis* and MtbΔ*sapM*Comp. However, no activity was obtained in MtbΔ*sapM*. Data is the mean (±SE) of 3 independent experiments carried out in triplicates. ***, *P*<0.001 (One-way ANOVA). (D) Growth kinetics of *M.tuberculosis*, MtbΔ*sapM* and MtbΔ*sapM*Comp in MB7H9 medium. Cultures were inoculated in duplicates with a starting absorbance (A_600 nm_) of 0.05 and the growth was monitored for 12 days. There was no significant difference in the growth of any strain. The values of absorbance are represented as the mean (±SE) of two independent experiments carried out in duplicates.

To evaluate the role of SapM in the physiology and pathogenesis of *M.tuberculosis*, we constructed a *sapM* mutant of *M.tuberculosis* (MtbΔ*sapM*). Disruption of *sapM* was confirmed by three different approaches. Firstly, we carried out PCR amplifications by employing the primers SapM-F and SapM-R (Fig. 1A). DNA from *M.tuberculosis* yielded a PCR product of 0.8 kb while the DNA from MtbΔ*sapM* resulted in a PCR product of 2.2 kb corresponding to replacement of a 500 bp region in the targeted gene by hygromycin resistance cassette (∼1.9 kb) (Fig. 1A). Secondly, the above amplification products were subjected to DNA sequencing that further confirmed the disruption of *sapM* in MtbΔ*sapM*. Thirdly, the disruption of *sapM* was further confirmed by immunoblot analysis by employing SapM specific polyclonal antibodies (Fig. 1B). In the case of *M.tuberculosis*, the immunoblot analysis by using the culture filtrate confirmed the presence of SapM as a 28 kDa band. This 28 kDa band was absent in the immunoblot analysis carried out with the culture filtrate from the mutant strain. Complementation of *sapM* in the case of MtbΔ*sapM*Comp was confirmed by the restoration of 28 kDa SapM band when the culture filtrate from this strain was subjected to immunoblot analysis.

Further, we evaluated the phosphatase activity of SapM in mycobacterial culture filtrates by measuring the conversion of *p*-nitrophenyl phosphate (pNPP) into *p-*nitrophenol (pNP). We detected the presence of phosphatase activity in the culture filtrate of *M.tuberculosis* (9 pmol/min/µg), while the activity of SapM was absent from the culture filtrate of MtbΔ*sapM* (Fig. 1C). However, the activity was restored when the MtbΔ*sapM* was complemented with *sapM* gene on a plasmid (35.9 pmol/min/µg) and was in fact, 4 fold higher than the parental strain. pSD7 plasmid used for the complementation has an origin of replication from *M.fortuitum* and is reported to maintain a copy number of 3 to 5 in mycobacteria [17], [24]. The higher activity of SapM in the culture filtrate of MtbΔ*sapM*Comp when compared with the activity observed in the culture filtrate of the parental strain may be attributable to the higher gene dosage due to copy number of the complementation plasmid [17].

In addition, when the growth characteristics of these *M.tuberculosis* strains were assessed under standard culture conditions in MB7H9 media, MtbΔ*sapM* displayed no significant difference in growth when compared with *M.tuberculosis* or MtbΔ*sapM*Comp (Fig. 1D). Thus, SapM was dispensable for *in vitro* growth of *M.tuberculosis* in MB7H9 medium.

### MtbΔ*sapM* Exhibits Attenuated Growth in Human THP-1 Macrophages

**Figure 2:**
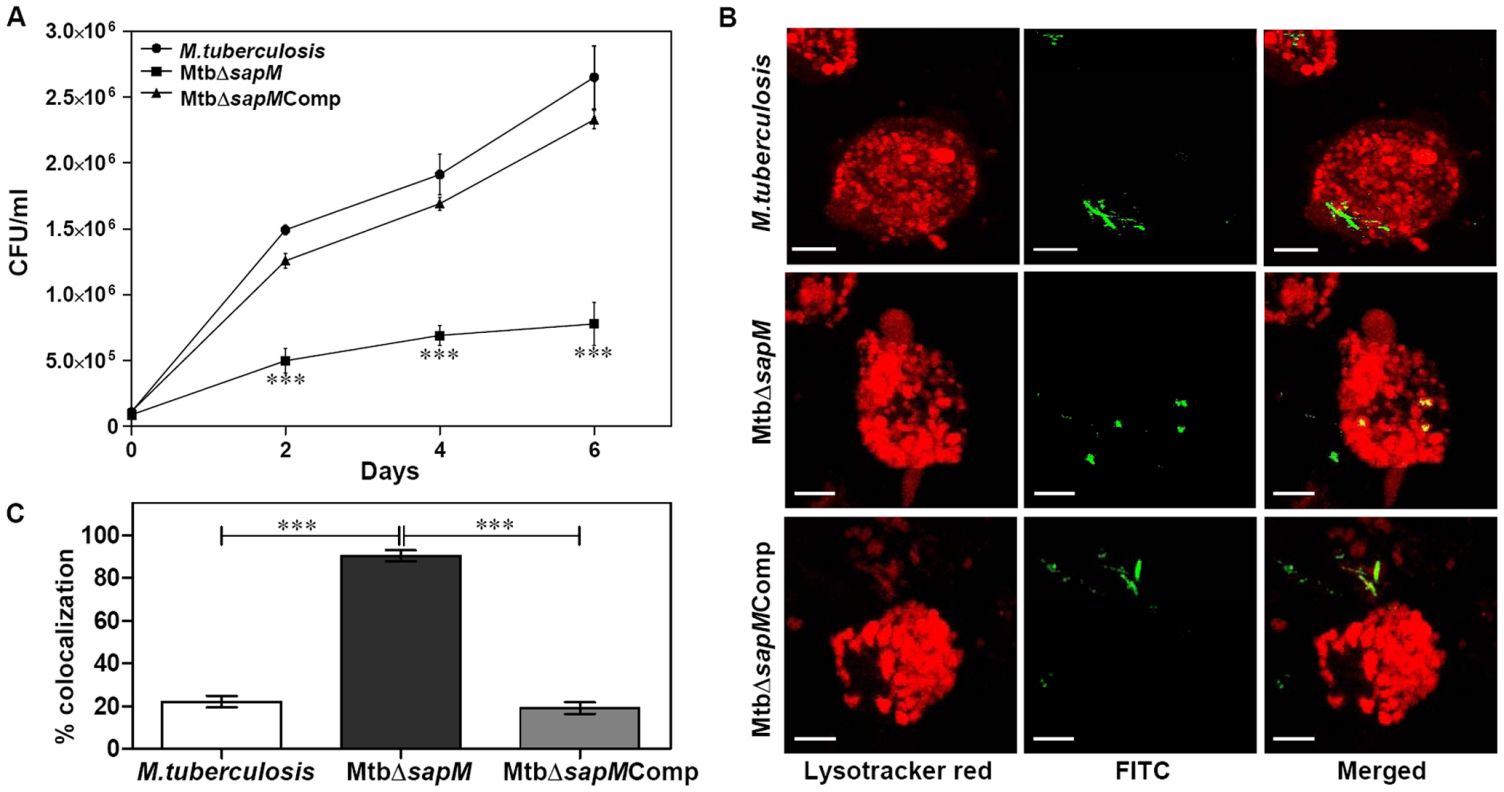
Disruption of *sapM* impairs growth of MtbΔ*sapM* in THP-1 macrophages and enhances phagosomal maturation. (A) Influence of *sapM* deletion on the growth of *M.tuberculosis* in THP-1 cells. THP-1 cells were infected with *M.tuberculosis*, MtbΔ*sapM* and MtbΔ*sapM*Comp separately at an MOI of 1∶3 (bacteria:macrophage). The number of intracellular viable bacteria were determined on each alternative day for 6 days. A significant attenuation in the growth of MtbΔ*sapM* in comparison to *M.tuberculosis* and MtbΔ*sapM*Comp was observed from second day post-infection. The growth of MtbΔ*sapM*Comp was comparable to *M.tuberculosis*. The values are represented as the mean (±SE) of three independent infections and the experiment was repeated three times. ***, *P*<0.001 (Two way ANOVA). (B) Increased localization of MtbΔ*sapM* in LysoTracker labeled compartments in THP-1 macrophages. Macrophages were infected with FITC labelled *M.tuberculosis*, MtbΔ*sapM* and MtbΔ*sapM*Comp (green) separately. After a 4 h incubation, the THP-1 cells were washed twice with fresh RPMI media and treated with 200 µg/ml amikacin for 2 h at 37°C to remove extracellular bacteria. Subsequently, the THP-1 cells were incubated with 50 nM Lysotracker red in RPMI (supplemented with 10% FBS) for 1 h. After this 7 h post infection period, the THP-1 cells were once washed with fresh RPMI media and fixed with 4% paraformaldehyde in PBS and observed under confocal microscope as described in the materials and methods. Representative fluorescent images depict that disruption of *sapM* lead to accumulation of MtbΔ*sapM* in acidified organelles (overlap of green and red images appears yellow) while the *M.tuberculosis* and MtbΔ*sapM*Comp were found in non-acidified organelles. The scale bars depict 5 µm. (C) Values indicate the percentage of phagosomes containing *M.tuberculosis*, MtbΔ*sapM* or MtbΔ*sapM*Comp that colocalized with Lysotracker red. On infecting the macrophages with MtbΔ*sapM*, it was observed that a significantly higher percentage of phagosomes containing mutant bacteria (∼86.57%) colocalized with LysoTracker red when compared with the phagosomes containing either *M.tuberculosis* (23.1%) or MtbΔ*sapM*Comp (22.72%). Data is the mean (±SE) of 3 independent experiments carried out in triplicates, with a minimum of 100 phagosomes counted per experiment for each sample. ***, *P*<0.001 (One way ANOVA).

Intracellular growth of *M.tuberculosis*, MtbΔ*sapM* and MtbΔ*sapM*Comp was compared in THP-1 macrophages. *M.tuberculosis* grew normally in THP-1 macrophages till 6 days post-infection. A significant difference was observed in the growth of MtbΔ*sapM* in comparison to the parental strain at 2 days post-infection corresponding to a 3 fold reduction in CFU. Further, the growth was followed up to 6 days and it was observed that the difference in the growth between MtbΔ*sapM* and the parental strain became wider with the passage of time. The growth of MtbΔ*sapM*Comp was similar to *M.tuberculosis* at 4 and 6 days post-infection (Fig. 2A). These results demonstrate the importance of *sapM* in the growth of the pathogen in the human THP-1 macrophages.

### SapM is Indispensable for Phagosomal Maturation Arrest in THP-1 Macrophages

We investigated the involvement of SapM in arresting phagosomal maturation by studying the colocalization of FITC labelled *M.tuberculosis* strains with LysoTracker Red, an acidotropic dye that freely permeates cell membranes and remains trapped in acidic compartments such as late endosomal and lysosomal organelles upon protonation [25], [26]. The FITC labeling protocol used in this study did not affect the survival of *M.tuberculosis* as similar bacterial CFU were obtained on plating the samples from FITC labeled and unlabelled bacteria (used as a control for labeling) on MB7H11 agar, at the end of the overnight incubation (data not shown). We observed that only 23.1% and 22.72% of FITC labeled *M.tuberculosis* and MtbΔ*sapM*Comp bacteria colocalized with LysoTracker rich compartments in THP-1 cells, respectively, indicating an efficient arrest of phagosomal maturation (Fig. 2B and 2C). In contrast, a significantly more colocalization of MtbΔ*sapM* (86.57%) was observed with LysoTracker demonstrating that the disruption of *sapM* impaired the ability of *M.tuberculosis* to arrest phagosomal maturation. The possibility that the phagosomal maturation observed in the case of MtbΔ*sapM* may be a result of attenuation in its growth was ruled out by determination of CFU from various samples. For this, at the time of proceeding for the fixation of THP-1 cells infected with either *M.tuberculosis* or MtbΔ*sapM* or MtbΔ*sapM*Comp with paraformaldehyde for evaluation under confocal microscope, the infected THP-1 cells from duplicate wells in each case were lysed and plated on MB7H11 agar for bacterial enumeration. We recovered a comparable number of CFU in all three cases. These results indicate that the mutation of *sapM* gene did not affect the growth of the pathogen after 7 h of infection and the inability of MtbΔ*sapM* to arrest the phagosomal maturation was due to the absence of SapM. These results demonstrate the importance of SapM in *M.tuberculosis* for the arrest of phagosomal maturation.

### Disruption of SapM Renders *M.tuberculosis* Severely Attenuated for Growth in Guinea Pig Tissues

**Figure 3:**
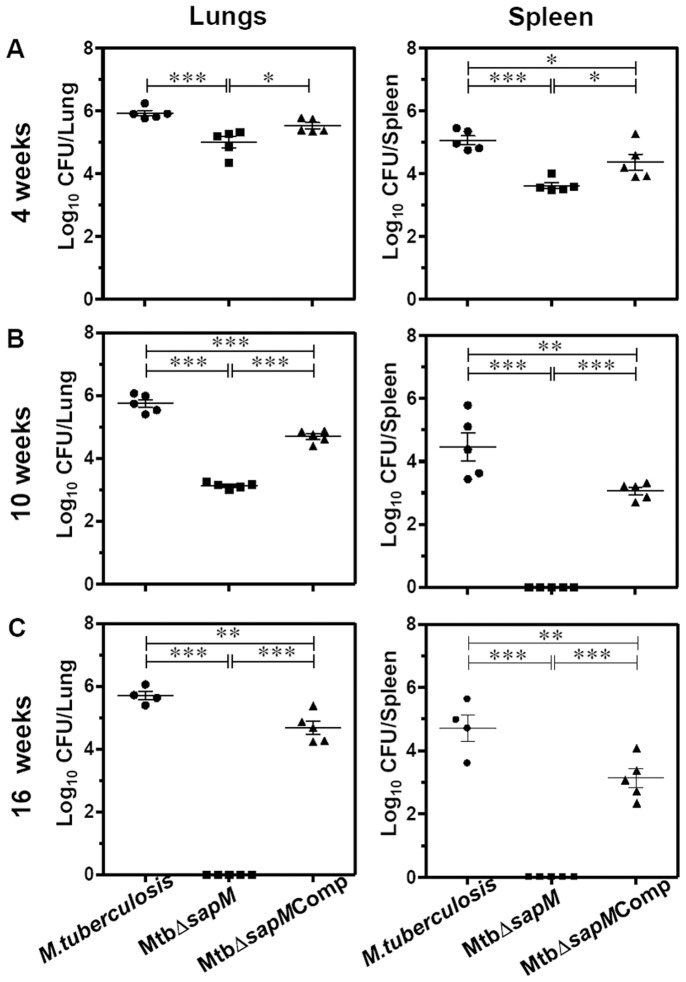
Disruption of *sapM* in *M.tuberculosis* leads to the attenuation of the pathogen in guinea pigs. The figure depicts bacillary load in the lungs and spleen of guinea pigs (n = 5) infected with *M.tuberculosis*, MtbΔ*sapM* and MtbΔ*sapM*Comp at (A) 4 weeks, (B) 10 weeks and (C) 16 weeks post-infection. Guinea pigs infected with MtbΔ*sapM* exhibited a significantly reduced bacillary load in the lungs as well as spleen when compared with the animals infected with either *M.tuberculosis* or MtbΔ*sapM*Comp. Each data point represents the Log_10_ CFU value for an individual animal and the bar depicts mean (±SE) for each group. Missing data points represent the animals that succumbed to disease before the time of euthanasia. *, *P*<0.05; **, *P*<0.01; ***, *P*<0.001 (One way ANOVA).

In order to elucidate the importance of SapM in the growth of *M.tuberculosis* in the host, we have employed guinea pig model of experimental tuberculosis. Guinea pigs were infected with 10–30 bacilli of *M.tuberculosis*, MtbΔ*sapM* or MtbΔ*sapM*Comp separately by using aerosol route of infection and euthanized at 4, 10 and 16 weeks post-infection. At 4 weeks post-infection, the bacillary load in the lungs of *M.tuberculosis* infected guinea pigs and MtbΔ*sapM*Comp infected guinea pigs corresponded to 5.93 log_10_ CFU and 5.53 log_10_ CFU, respectively. However, the bacillary load in the lungs of MtbΔ*sapM* infected guinea pigs was significantly lower (5.0 log_10_ CFU) which was 0.93 log_10_ CFU and 0.53 log_10_ CFU less than the bacillary load in the lungs of *M.tuberculosis* infected animals (*P*<0.001) and MtbΔ*sapM*Comp infected animals (*P*<0.05), respectively (Fig. 3A). At this time point, the spleen of *M.tuberculosis* infected guinea pigs and MtbΔ*sapM*Comp infected guinea pigs exhibited a bacillary load of 5.06 log_10_ CFU and 4.37 log_10_ CFU, respectively. The splenic bacillary load in MtbΔ*sapM* infected guinea pigs was significantly less (3.62 log_10_ CFU) when compared with the splenic bacillary load in *M.tuberculosis* infected guinea pigs (1.44 log_10_ CFU fewer bacilli, *P*<0.001) or MtbΔ*sapM*Comp infected guinea pigs (0.75 log_10_ CFU fewer bacilli, *P*<0.05) (Fig. 3A).

At 10 weeks post-infection, the bacillary load in the lungs of *M.tuberculosis* infected animals and MtbΔ*sapM*Comp infected animals corresponded to 5.75 log_10_ CFU and 4.7 log_10_ CFU, respectively. However, the MtbΔ*sapM* infected animals exhibited a significantly reduced pulmonary bacillary load (3.14 log_10_ CFU) as compared to *M.tuberculosis* infected animals (2.61 log_10_ CFU fewer bacilli, *P*<0.001) and MtbΔ*sapM*Comp infected animals (1.56 log_10_ CFU fewer bacilli, *P*<0.001) (Fig. 3B). The infection of guinea pigs with *M.tuberculosis* or MtbΔ*sapM*Comp resulted in a splenic bacillary load of 4.47 log_10_ CFU and 3.06 log_10_ CFU, respectively. However, at this time point, we did not recover any bacilli from the spleens of guinea pigs infected with MtbΔ*sapM* (Fig. 3B).

When the post-challenge period was extended to 16 weeks, the bacillary load in the lungs of *M.tuberculosis* infected animals and MtbΔ*sapM*Comp infected animals corresponded to 5.7 log_10_ CFU and 4.69 log_10_ CFU, respectively. However, at this time point no MtbΔ*sapM* bacilli were recovered from the lungs of the infected animals (Fig. 3C). The animals infected with *M.tuberculosis* or with MtbΔ*sapM*Comp exhibited a splenic bacillary load of 4.72 log_10_ CFU and 3.14 log_10_ CFU, respectively. As observed at 10 weeks time point, no bacilli were recovered from the spleens of the guinea pigs infected with MtbΔ*sapM* (Fig. 3C). The complete absence of bacilli from the lungs and spleens of the animals infected with MtbΔ*sapM* at both 10 and 16 weeks post-infection demonstrates that SapM plays an important role in the growth of *M.tuberculosis* in the host.

### Disruption of *sapM* Results in a Significantly Reduced Gross Pathological Damage

We assessed the influence of disruption of *sapM* on the ability of *M.tuberculosis* to cause disease and pathology. The trend observed in pulmonary and splenic bacillary load was substantiated by the gross pathological changes. At 4 weeks post-infection, the lungs of the animals infected with either *M.tuberculosis* or MtbΔ*sapM*Comp displayed moderate involvement with occasional large tubercles. The lungs of the animals infected with MtbΔ*sapM* were inflamed and displayed small-sized tubercles. The gross pathological scores for the lungs of MtbΔ*sapM* infected guinea pigs were significantly less (*P*<0.05) than the corresponding scores in *M.tuberculosis* or MtbΔ*sapM* Comp infected animals. The hepatic and splenic tissues of animals infected with any of the *M.tuberculosis* strains exhibited moderate involvement with predominantly scanty and extremely small necrotic lesions. No significant differences were observed in the gross pathological scores for liver and spleen of guinea pigs infected with *M.tuberculosis*, MtbΔ*sapM* or MtbΔ*sapM*Comp (data not shown).

**Figure 4:**
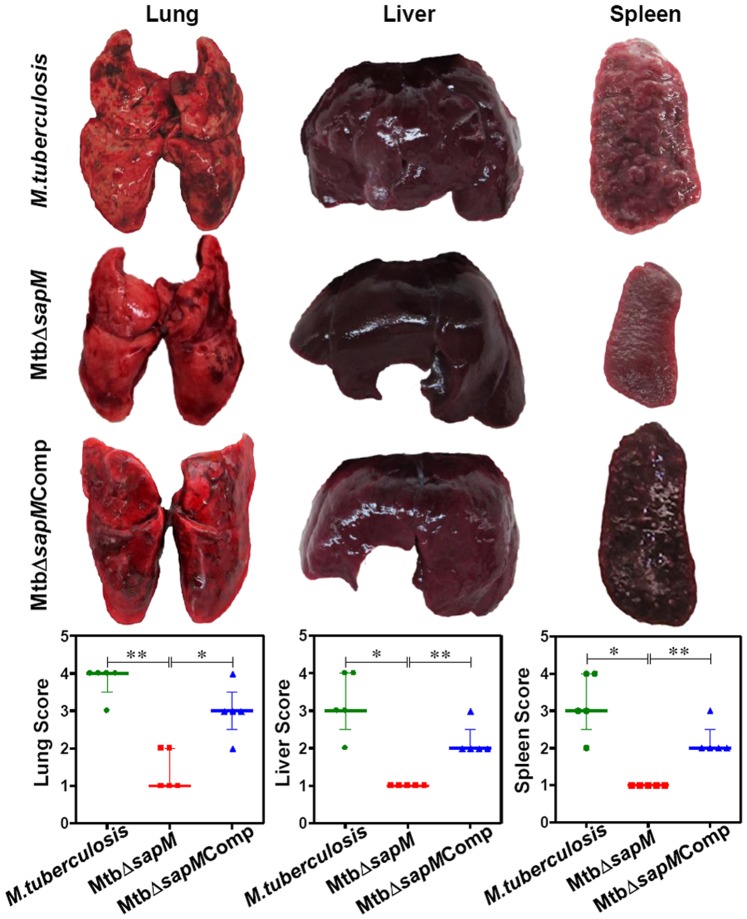
Gross pathology of guinea pig organs infected with variou*s M.tuberculosis* strains at 10 weeks. The figure depicts representative photographs of gross pathological lesions and graphical depiction of gross scores of lung, liver and spleen of guinea pigs (n = 5) infected with *M.tuberculosis*, MtbΔ*sapM* and MtbΔ*sapM*Comp euthanized at 10 weeks post-infection. Each data point represents the score of an individual animal, and the bars depict medians (±interquartile range) for each group. Organs of the MtbΔ*sapM* infected animals exhibited fewer and smaller pulmonary, hepatic and splenic lesions when compared with the organs of guinea pigs infected with either *M.tuberculosis* or the MtbΔ*sapM*Comp. *, *P*<0.05; **, *P*<0.01 (Mann-Whitney U test).

At 10 weeks post-infection, the animals infected with either *M.tuberculosis* or MtbΔ*sapM*Comp exhibited enhanced pathological damage in the organs than at 4 weeks post-infection (Fig. 4). The guinea pigs infected with *M.tuberculosis* exhibited extensive involvement of lungs with the presence of numerous large and small tubercles. Numerous small-sized tubercles were observed in the liver while the spleens were characterized by enlargement in size with numerous large coalescing tubercles and occasional attrition of capsular structure. The organs of MtbΔ*sapM*Comp infected animals also exhibited extensive involvement with numerous large sized tubercles effacing the entire organs. Noticeably, MtbΔ*sapM* infected guinea pigs exhibited a significantly reduced pathological damage in comparison to the *M.tuberculosis* or MtbΔ*sapM*Comp infected guinea pigs. Predominantly scanty and small tubercles were observed in the lungs of animals infected with MtbΔ*sapM* when compared with the lungs of the animals infected with *M.tuberculosis* (*P*<0.01) or MtbΔ*sapM*Comp (*P*<0.05). Moreover, liver and spleen of MtbΔ*sapM* infected animals also exhibited a significant reduction in the pathological damage as compared to *M.tuberculosis* (*P*<0.05) or MtbΔ*sapM*Comp infected animals (*P*<0.01) (Fig. 4).

**Figure 5:**
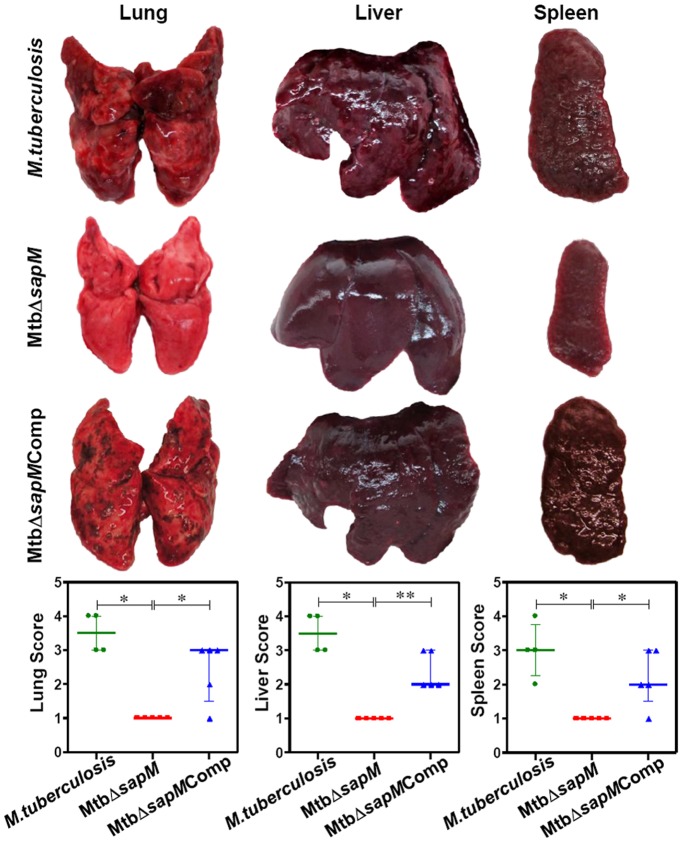
Gross pathology of guinea pig organs infected with various *M.tuberculosis* strains at 16 weeks. The figure depicts representative photographs of gross pathological lesions and graphical depiction of gross scores of lung, liver and spleen of guinea pigs (n = 5) infected with *M.tuberculosis*, MtbΔ*sapM* and MtbΔ*sapM*Comp euthanized at 16 weeks post-infection. Each data point represents the score of an individual animal, and the bars depict medians (±interquartile range) for each group. Organs of the MtbΔ*sapM* infected animals exhibited minimal involvement with the presence of only a few visible tubercles when compared with the organs of guinea pigs infected with either *M.tuberculosis* or the MtbΔ*sapM*Comp that exhibited a heavy involvement with numerous large sized tubercles and necrosis. *, *P*<0.05; **, *P*<0.01 (Mann-Whitney U test). Missing data points represent the animals that succumbed to disease before the time of euthanasia.

At 16 weeks post-infection, 1 out of 5 animals infected with *M.tuberculosis* succumbed to the disease while the remaining animals displayed extensive pathological damage (Fig. 5). The lungs of these animals were edematous, characterized by numerous large sized coalescing and suppurative tubercles distributed throughout the lungs. The *M.tuberculosis* infected animals were characterized by extensive involvement of liver with numerous large tubercles and splenomegaly. In addition, scattered areas of necrosis were observed both in the liver and spleen. The lungs, liver, and spleen of guinea pigs infected with MtbΔ*sapM*Comp exhibited heavy involvement with the presence of numerous large tubercles. Noticeably, the organs of MtbΔ*sapM* infected guinea pigs displayed a minimal involvement in comparison to the organs of *M.tuberculosis* infected guinea pigs (*P*<0.05) with presence of only a few visible tubercles and no manifestation of tissue damage. The gross pathological scores of organs from the MtbΔ*sapM* infected animals were significantly less in comparison to the corresponding scores from MtbΔ*sapM*Comp infected animals [lungs (*P*<0.05), liver (*P*<0.01) and spleen (*P*<0.05)] (Fig. 5). Hence, the pathological damage observed in the organs of animals infected with various strains was in agreement with the bacillary load recovered from the organs.

### MtbΔ*sapM* Displays a Diminution in Granulomatous Inflammation

Histopathological analysis of lung and liver sections further substantiated the gross pathological observations. At 4 weeks post-infection, guinea pigs infected with *M.tuberculosis* or MtbΔ*sapM*Comp exhibited scattered areas of granulomatous inflammation encompassing ∼36% and ∼28% of the lung section, respectively, with no significant differences observed between these two groups. However, the lungs of MtbΔ*sapM* infected animals exhibited a significant decrease (*P*<0.05) in the pathological damage in comparison to the lungs of the *M.tuberculosis* infected animals encompassing ∼17% of lung area. No significant differences were observed amongst the livers of the infected animals belonging to any of the groups (data not shown).

**Figure 6:**
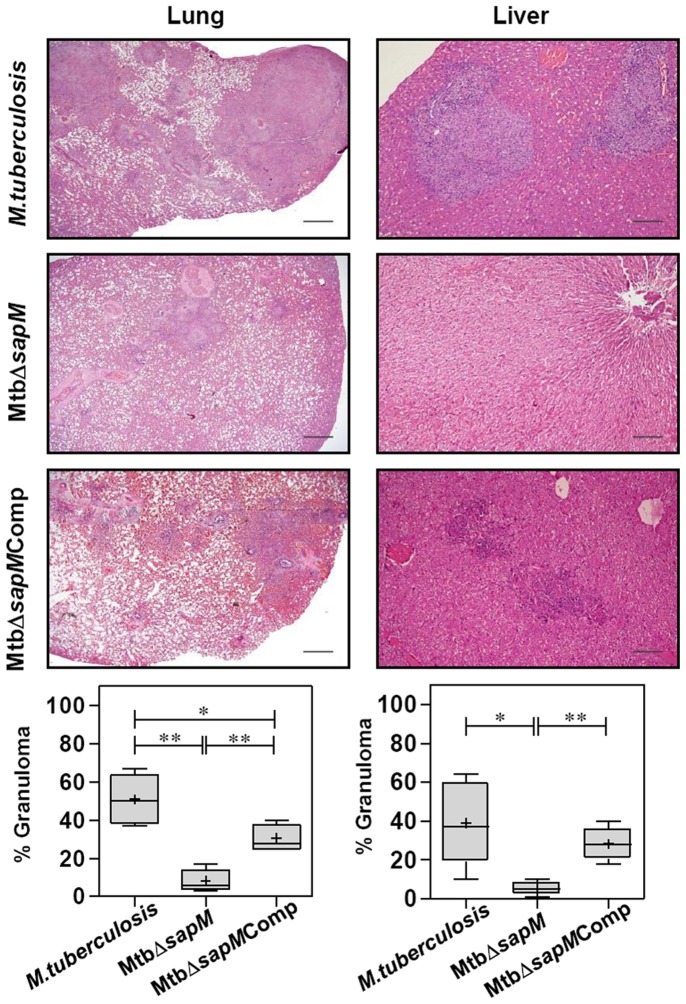
Histopathology of guinea pig organs infected with variou*s M.tuberculosis* strains at 10 weeks. The figure depicts representative lower magnification (20x) photomicrographs of H&E stained 5 µm sections of lung and liver of guinea pigs (n = 5) infected with *M.tuberculosis*, MtbΔ*sapM* and MtbΔ*sapM*Comp euthanized at 10 weeks post-infection. Pulmonary and hepatic granuloma consolidations were graphically represented as % granuloma by box plot (median values are denoted by horizontal line, the mean is represented by ‘+’, inter quartile range by boxes, and the maximum and minimum values by whiskers). MtbΔ*sapM* infected animals displayed a significant diminution in granulomatous infiltration in the lungs and liver when compared with numerous coalescing granulomas observed in the organs of animals infected with *M.tuberculosis* or MtbΔ*sapM*Comp. The scale bars depict 500 μm. *, *P*<0.05; **, *P*<0.01 (Mann-Whitney U test).

At 10 weeks post-infection, animals infected with *M.tuberculosis* exhibited numerous coalescing granulomas, covering ∼50% area of the lung sections (Fig. 6). In the animals infected with MtbΔ*sapM*Comp, foci of numerous granulomatous lesions were observed encompassing ∼30% of the lung area. However, the granulomatous inflammation in MtbΔ*sapM* infected animals was significantly less (*P*<0.01), corresponding to only ∼8% lung consolidation due to the presence of few small and discrete granulomas, when compared with *M.tuberculosis* or MtbΔ*sapM*Comp infected animals. The lungs of the MtbΔ*sapM* infected animals, showed well-preserved alveolar spaces with only a few scattered areas of diffused infiltration. The pathological changes in the liver of animals infected with MtbΔ*sapM* displayed a significantly reduced granulomatous infiltration (∼5.2%) when compared with *M.tuberculosis* (∼39%, *P*<0.05) or MtbΔ*sapM*Comp (∼28%, *P*<0.01) infected animals (Fig. 6).

**Figure 7:**
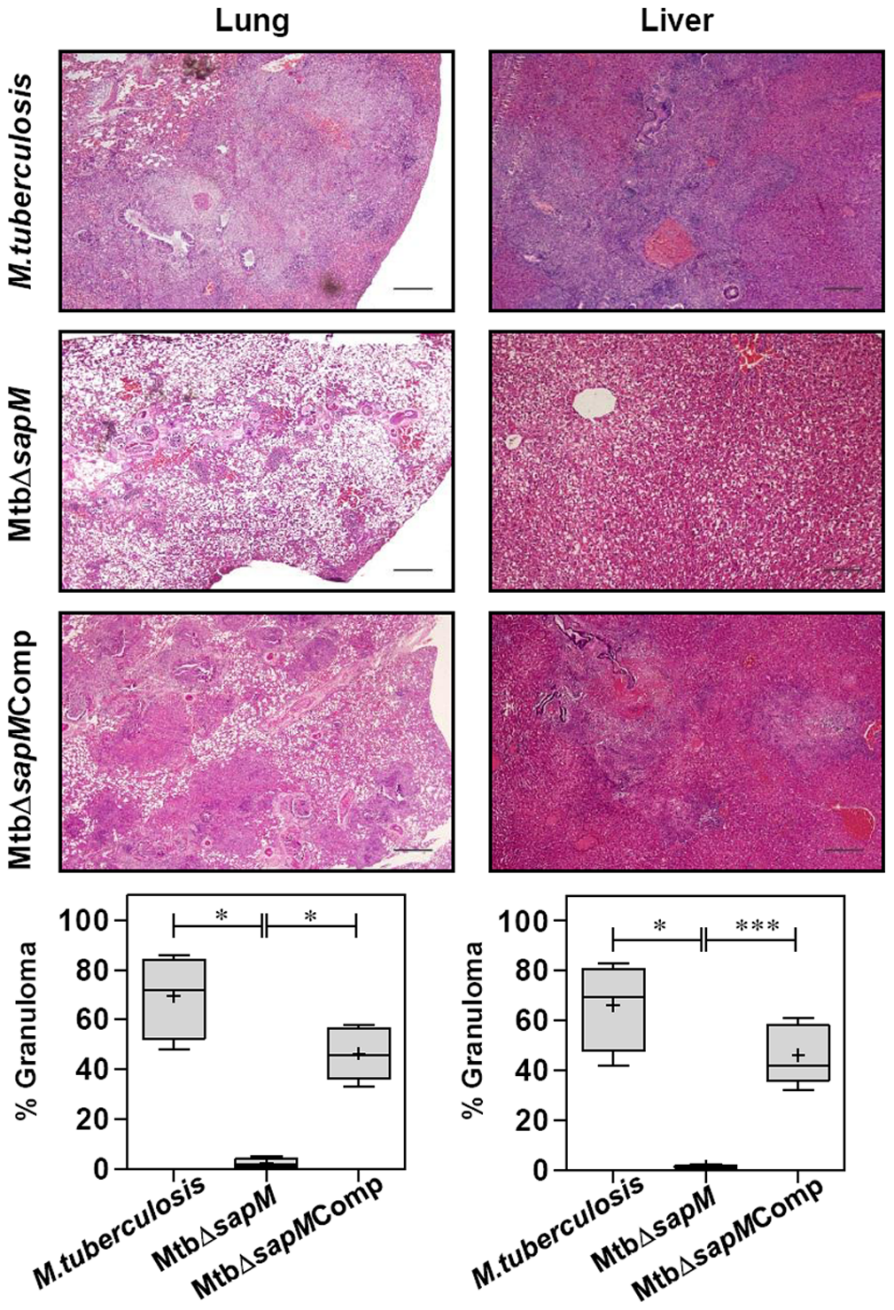
Histopathology of guinea pig organs infected with various *M.tuberculosis* strains at 16 weeks. The figure depicts representative lower magnification (20x) photomicrographs of H&E stained 5 µm sections of lung and liver of guinea pigs (n = 5) infected with *M.tuberculosis*, MtbΔ*sapM* and MtbΔ*sapM*Comp euthanized at 16 weeks post-infection. Pulmonary and hepatic granuloma consolidations were graphically represented as % granuloma by box plot (median values are denoted by horizontal line, the mean is represented by ‘+’, inter quartile range by boxes, and the maximum and minimum values by whiskers). While the lungs and liver of *M.tuberculosis* or MtbΔ*sapM*Comp infected guinea pigs exhibited large areas of granulomatous inflammation, the animals infected with MtbΔ*sapM* exhibited normal pulmonary and hepatic parenchyma. The scale bars depict 500 μm. *, *P*<0.05; ***, *P*<0.001 (Mann-Whitney U test). Missing data points represent the animals that succumbed to disease before the time of euthanasia.

At 16 weeks post-infection, the animals infected with *M.tuberculosis* exhibited multiple coalescing foci of necrotic granulomas (∼70%) effacing the pulmonary parenchyma (Fig. 7). Lungs of the animals infected with MtbΔ*sapM*Comp displayed ∼46% lung consolidation. In these groups, extensive necrosis resulted in the loss of lung micro-architecture. In contrast, the pulmonary tissue organization was restored in the animals infected with MtbΔ*sapM* with negligible pathological damage in comparison to *M.tuberculosis* or MtbΔ*sapM*Comp infected animals (∼2.4% granuloma, *P*<0.05). On comparing the pathological changes in liver, extensive granulomatous infiltration in the hepatic lobules was observed in guinea pigs infected with either *M.tuberculosis* (∼66%) or MtbΔ*sapM*Comp (∼46%). Negligible granulomatous infiltration (∼1.2%) was observed in the case of MtbΔ*sapM* infected animals, in comparison to the liver of guinea pigs infected with *M.tuberculosis* (*P*<0.05) or MtbΔ*sapM*Comp (*P*<0.001). Architecture of hepatic lobules was completely preserved in the case of MtbΔ*sapM* infected animals (Fig. 7). The negligible histopathological damage in the tissues of the animals infected with MtbΔ*sapM* demonstrates that SapM is crucial for the pathogenesis of *M.tuberculosis* in the host tissues.

### Guinea Pigs Infected with *sapM* Mutant of *M.tuberculosis* Exhibit a Longer Survival Time

**Figure 8:**
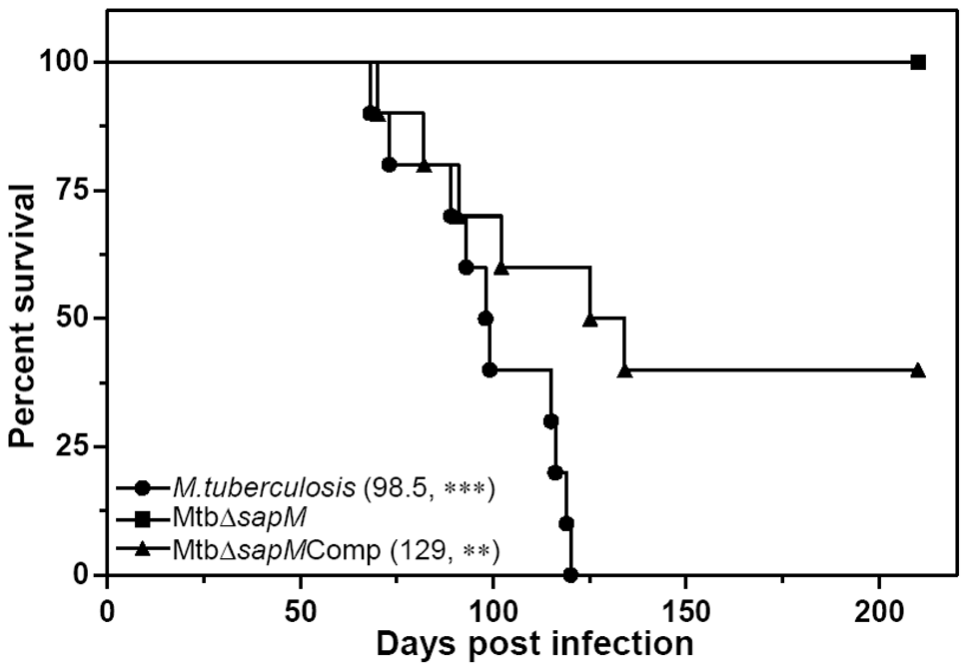
Influence of disruption of *sapM* gene of *M.tuberculosis* on the survival of guinea pigs post-infection. Guinea pigs aerogenically infected with 10–30 bacilli of either *M.tuberculosis*, MtbΔ*sapM* or MtbΔ*sapM*Comp were monitored for survival (n = 10) up to 210 days post-infection. In case, the animals were infected with MtbΔ*sapM*, 100% of the animals survived. While no animal survived in the *M.tuberculosis* infected group, 40% survivors were observed in case the animals were infected with MtbΔ*sapM*Comp. The median survival time for each infected group is mentioned in brackets. * represents the significant difference in comparison to the MtbΔ*sapM*. **, *P*<0.01; ***, *P*<0.001 [Log-rank (Mantel-Cox) Test].

In addition to the differences observed in the bacterial burden and pathological damage in the tissues of infected guinea pigs, a significant difference was observed in the survival of the infected animals. All the guinea pigs infected with the parental strain succumbed to death within 120 days of infection, with a median survival time (MST) of 98.5 days (Fig. 8). In the case of infection with MtbΔ*sapM*Comp, 60% of the animals died during the course of the experiment with a MST of 129 days. In contrast, no deaths were observed in the case of animals infected with MtbΔ*sapM* up to 210 days post-infection, after which the experiment was terminated. Thus, the survival time of guinea pigs infected with MtbΔ*sapM* mutant was significantly longer than the animals infected with *M.tuberculosis* (*P*<0.001) or MtbΔ*sapM*Comp (*P*<0.05).

## Discussion

*Mycobacterium tuberculosis* is one of the most successful intracellular parasitic bacteria. When aerosol containing *M.tuberculosis* is inhaled by guinea pigs, the pathogen reaches lungs and is ingested by the resident alveolar macrophages. Macrophages are equipped with a plethora of antimicrobial mechanisms to kill pathogens [27], [28], [29]. However, the success of *M.tuberculosis* as a highly adapted human pathogen has largely been attributed to its ability to survive successfully in the infected macrophages [4], [30]. *M.tuberculosis* blocks the biogenesis of phagolysosome, the very organelle responsible for the routine elimination of microorganisms by phagocytic cells [31], [32]. This strategy employed to arrest phagosomal maturation helps *M.tuberculosis* prevent its killing inside the host macrophage. In fact, by employing a genetic screen it has been demonstrated that *M.tuberculosis* mutants that are defective in the arrest of phagosome maturation show a reduced intracellular survival [33]. Hence, *M.tuberculosis* proteins and lipids involved in the phagosome maturation blockage hold great promise as a target for the design of anti-tubercular molecules.

Previous studies have reported divergent observations with respect to the role of SapM in phagosomal maturation arrest in mycobacteria [11], [13], [14]. Hence, in this study, we first attempted to reascertain the involvement of SapM in phagosomal maturation arrest in *M.tuberculosis* and also evaluated the influence of *sapM* mutation on the growth of the pathogen in macrophages. Further, for the first time, we have also evaluated the importance of SapM in the pathogenesis of *M.tuberculosis* by conducting animal studies with a *sapM* mutant along with the parental strain.

Our results demonstrate that *sapM* is dispensable for the *in vitro* growth of *M.tuberculosis* in the broth culture. However, the growth kinetics of MtbΔ*sapM* in human THP-1 macrophages up to 6 days post-infection revealed an attenuated growth phenotype when compared with the parental strain. Further, to reascertain the involvement of SapM in the arrest of phagosomal maturation in *M.tuberculosis*, we carried out colocalization studies. On examining the colocalization of FITC labeled *M.tuberculosis* containing phagosomes with Lysotracker, we observed that while *M.tuberculosis* primarily resides in non-acidified compartments of THP-1 cell line, a mutation in *sapM* significantly increased the number of *M.tuberculosis* in the acidified compartments. Thus, by clearly demonstrating the inability of MtbΔ*sapM* to arrest phagosomal maturation and its reversal by MtbΔ*sapM*Comp, our studies reconfirm the involvement of SapM in arresting the maturation of phagosomes in *M.tuberculosis* and are in agreement with the conclusion drawn by Vergne *et al.* and Saikolappan *et al.* who reported that SapM is important for arresting phagosomal maturation in *M.tuberculosis* [11], [13]. However, Festjens *et al.*, by conducting colocalization experiments by employing Mf4/4 murine macrophages infected with either BCG or its *sapM* mutant reported that SapM was not necessary for the arrest of phagosome maturation in BCG [14]. We do not, at present, understand the reason behind the different observations on the involvement of SapM in arresting the phagosomal maturation in *M.tuberculosis* and BCG, however, it is noteworthy that phagosomal maturation and its arrest are complex processes with multifactorial requirement [7]. A number of studies have implicated several mycobacterial proteins in phagosomal maturation arrest such as lipoarabinomannan (LAM), trehalose dimycolate (TDM), a serine/threonine kinase (PknG), a lipoamide dehydrogenase (LpdC), a Zn^2+^ metalloprotease (Zmp1), tyrosine phosphatase (PtpA) and Esx-1 secretion system [7], [34], [35], [36], [37], [38], [39], [40]. More importantly, it is becoming apparent that the requirement for these processes in all mycobacterial species may not necessarily be identical [36], [40]. By employing an *M.marinum* mutant that was able to produce both, early secretory antigenic 6 kDa (ESAT-6) and culture filtrate protein 10 (CFP-10), but was unable to secrete these proteins, it was shown by Tan *et al.* that on infecting the macrophages, a much higher percentage of phagosomes containing mutant bacteria (∼72%) colocalized with LysoTracker when compared with the phagosomes containing the parental *M.marinum* clearly implicating ESAT-6 and CFP-10 in phagosomal maturation arrest in this mycobacterial species [40]. ESAT-6/CFP-10 are encoded by the RD1 locus [40]. However, BCG was able to arrest phagosomal maturation inspite of the absence of RD1 locus, meaning thereby that phagosomal maturation in the case of BCG can be arrested without ESAT-6 and CFP-10 although these proteins are necessary for this arrest in *M.marinum* [6], [25], [40]. Thus, these observations suggest that phagosomal maturation arrest in BCG, may involve the set of proteins which may not be identical with that employed by *M.tuberculosis* or *M.marinum* [6], [25]. SapM is involved in phagosomal maturation arrest in *M.tuberculosis* as shown by Vergne *et al.*, Saikolappan *et al.* and reconfirmed by our study [11], [13]. However, it is possible that the absence of SapM, like the absence of ESAT-6/CFP-10, may not influence the phagosomal maturation arrest in BCG as observed by Festjens *et al.* due to involvement of other proteins [14].

The most substantial evidence for the role of SapM in *M.tuberculosis* pathogenesis emerged from our studies in the guinea pig model of infection. Guinea pig model of experimental tuberculosis has been considered as one of the most relevant animal models [41]. It has been established that following the pulmonary infection, the course of disease including bacillemia and hematogenous reseeding of the lungs, in guinea pigs is similar to that in humans [41]. After the pathogen disseminates from lungs to the pulmonary lymph nodes via hematogenous spread, it appears in spleens ∼3 weeks post-infection [42]. The bacilli start reseeding in the lung by ∼4 weeks to form secondary granulomas. Moreover, the extreme susceptibility of guinea pigs to *M.tuberculosis* infection and high degree of similarity with humans in the symptoms and pathophysiology, substantiate the use of this animal model to illuminate the events in the pathogenesis of pulmonary TB [43].

Our study demonstrated that while *M.tuberculosis* exhibited normal growth in the organs of guinea pigs, the growth of MtbΔ*sapM* in the organs of infected guinea pigs was highly attenuated. In fact, at the end of 16 weeks, no mycobacteria were recovered from the lungs or spleens of MtbΔ*sapM* infected animals. *M.tuberculosis* and MtbΔ*sapM*Comp exhibited normal growth in the guinea pig organs although the growth of MtbΔ*sapM*Comp was a bit less than *M.tuberculosis* at the end of 16 weeks post-infection. Thus, for the first time our observations demonstrate that SapM is indispensable for the growth of *M.tuberculosis* in the host which is further substantiated by the observations that guinea pigs infected with MtbΔ*sapM* exhibited a significantly reduced pathological damage as compared to the animals infected with *M.tuberculosis*.

Survival of infected animals is one of the best parameters to evaluate the involvement of a gene in the pathogenesis of an organism [44], [45]. Hence, we evaluated the effect of the disruption of *sapM* on the survival of the infected animals. The animals infected with *M.tuberculosis* gradually succumbed to death within 120 days post-infection with a MST of 98.5 days. MtbΔ*sapM*Comp infected guinea pigs also exhibited comparable survival time with a MST of 129 days. However, the influence of the deletion of *sapM* gene on the survival of the animals was unambiguous as during the total duration of the experiment (210 days) not even a single MtbΔ*sapM* infected animal succumbed to death. This is the most substantial evidence for the role of SapM in the pathogenesis of *M.tuberculosis*. Thus in the current study, we have demonstrated the importance of SapM in arresting the phagosomal maturation as well as in the pathogenesis of *M.tuberculosis*, establishing it as an important target for the development of new anti-tubercular molecules.

To summarize, we demonstrate that SapM mediates an important role in the protection of *M.tuberculosis* against the host defense by subverting the phagosomal maturation pathway. Disruption of *sapM* in *M.tuberculosis* resulted in a highly attenuated strain with an impaired ability to grow in the THP-1 macrophages as well as in the guinea pig tissues. Thus, our studies establish SapM as a potential drug target. The fact that there are no known human analogues of SapM makes it even more important target for the development of new therapeutic molecules against TB. In addition, the secretory nature of SapM presents a unique opportunity in order to avoid the drug permeability issue due to thick hydrophobic cell envelope of *M.tuberculosis* [46].

## References

[1] WHO (2012) Tuberculosis Global Facts 2011/2012. Available: http://www.who.int/tb/publications/2011/factsheet_tb_2011.pdf. Accessed 2013 May 6.

[2] WHO (2011) Tuberculosis MDR-TB & XDR-TB 2011. Progress Report. Available: http://www.who.int/tb/challenges/mdr/factsheet_mdr_progress_march2011.pdf. Accessed 2013 May 6.

[3] Li W, Xie J (2011) Role of mycobacteria effectors in phagosome maturation blockage and new drug targets discovery. J Cell Biochem 112: 2688–2693. 21678466 10.1002/jcb.23218

[4] de Chastellier C (2009) The many niches and strategies used by pathogenic mycobacteria for survival within host macrophages. Immunobiology 214: 526–542. 19261352 10.1016/j.imbio.2008.12.005

[5] Vergne I, Chua J, Singh SB, Deretic V (2004) Cell biology of *Mycobacterium tuberculosis* phagosome. Annu Rev Cell Dev Biol 20: 367–394. 15473845 10.1146/annurev.cellbio.20.010403.114015

[6] Via LE, Deretic D, Ulmer RJ, Hibler NS, Huber LA, et al (1997) Arrest of mycobacterial phagosome maturation is caused by a block in vesicle fusion between stages controlled by rab5 and rab7. J Biol Chem 272: 13326–13331. 9148954 10.1074/jbc.272.20.13326

[7] Philips JA (2008) Mycobacterial manipulation of vacuolar sorting. Cell Microbiol 10: 2408–2415. 18783482 10.1111/j.1462-5822.2008.01239.x

[8] Vieira OV, Botelho RJ, Grinstein S (2002) Phagosome maturation: aging gracefully. Biochem J 366: 689–704. 12061891 10.1042/BJ20020691 PMC1222826

[9] Fratti RA, Backer JM, Gruenberg J, Corvera S, Deretic V (2001) Role of phosphatidylinositol 3-kinase and Rab5 effectors in phagosomal biogenesis and mycobacterial phagosome maturation arrest. J Cell Biol 154: 631–644. 11489920 10.1083/jcb.200106049 PMC2196432

[10] Vieira OV, Botelho RJ, Rameh L, Brachmann SM, Matsuo T, et al (2001) Distinct roles of class I and class III phosphatidylinositol 3-kinases in phagosome formation and maturation. J Cell Biol 155: 19–25. 11581283 10.1083/jcb.200107069 PMC2150784

[11] Vergne I, Chua J, Lee HH, Lucas M, Belisle J, et al (2005) Mechanism of phagolysosome biogenesis block by viable *Mycobacterium tuberculosis* . Proc Natl Acad Sci U S A 102: 4033–4038. 15753315 10.1073/pnas.0409716102 PMC554822

[12] Saleh MT, Belisle JT (2000) Secretion of an acid phosphatase (SapM) by *Mycobacterium tuberculosis* that is similar to eukaryotic acid phosphatases. J Bacteriol 182: 6850–6853. 11073936 10.1128/jb.182.23.6850-6853.2000 PMC111434

[13] Saikolappan S, Estrella J, Sasindran SJ, Khan A, Armitige LY, et al (2012) The *fbpA/sapM* double knock out strain of *Mycobacterium tuberculosis* is highly attenuated and immunogenic in macrophages. PLoS One 7: e36198. 22574140 10.1371/journal.pone.0036198 PMC3344844

[14] Festjens N, Bogaert P, Batni A, Houthuys E, Plets E, et al (2011) Disruption of the SapM locus in *Mycobacterium bovis* BCG improves its protective efficacy as a vaccine against *M. tuberculosis* . EMBO Mol Med 3: 222–234. 21328541 10.1002/emmm.201000125 PMC3377067

[15] van Kessel JC, Hatfull GF (2007) Recombineering in *Mycobacterium tuberculosis* . Nat Methods 4: 147–152. 17179933 10.1038/nmeth996

[16] Bardarov S, Bardarov S Jr, Pavelka MS Jr, Sambandamurthy V, Larsen M, et al (2002) Specialized transduction: an efficient method for generating marked and unmarked targeted gene disruptions in *Mycobacterium tuberculosis*, *M. bovis* BCG and *M. smegmatis* . Microbiology 148: 3007–3017. 12368434 10.1099/00221287-148-10-3007

[17] Das Gupta SK, Bashyam MD, Tyagi AK (1993) Cloning and assessment of mycobacterial promoters by using a plasmid shuttle vector. J Bacteriol 175: 5186–5192. 8349558 10.1128/jb.175.16.5186-5192.1993 PMC204986

[18] Reddy PV, Puri RV, Khera A, Tyagi AK (2012) Iron storage proteins are essential for the survival and pathogenesis of *Mycobacterium tuberculosis* in THP-1 macrophages and the guinea pig model of infection. J Bacteriol 194: 567–575. 22101841 10.1128/JB.05553-11 PMC3264086

[19] Gelfand JA, Fauci AS, Green I, Frank MM (1976) A simple method for the determination of complement receptor-bearing mononuclear cells. J Immunol 116: 595–599. 815430

[20] Kudo K, Sano H, Takahashi H, Kuronuma K, Yokota S, et al (2004) Pulmonary collectins enhance phagocytosis of *Mycobacterium avium* through increased activity of mannose receptor. J Immunol 172: 7592–7602. 15187139 10.4049/jimmunol.172.12.7592

[21] Esteban MA, Mulero V, Munoz J, Meseguer J (1998) Methodological aspects of assessing phagocytosis of *Vibrio anguillarum* by leucocytes of gilthead seabream (*Sparus aurata* L.) by flow cytometry and electron microscopy. Cell Tissue Res 293: 133–141. 9634605 10.1007/s004410051105

[22] Jain R, Dey B, Dhar N, Rao V, Singh R, et al (2008) Enhanced and enduring protection against tuberculosis by recombinant BCG-Ag85C and its association with modulation of cytokine profile in lung. PLoS One 3: e3869. 19052643 10.1371/journal.pone.0003869 PMC2586085

[23] Mitchison DA, Wallace JG, Bhatia AL, Selkon JB, Subbaiah TV, et al (1960) A comparison of the virulence in guinea-pigs of South Indian and British tubercle bacilli. Tubercle 41: 1–22. 14423002 10.1016/s0041-3879(60)80019-0

[24] Bachrach G, Colston MJ, Bercovier H, Bar-Nir D, Anderson C, et al (2000) A new single-copy mycobacterial plasmid, pMF1, from *Mycobacterium fortuitum* which is compatible with the pAL5000 replicon. Microbiology 146 (Pt 2): 297–303. 10.1099/00221287-146-2-297 10708368

[25] Via LE, Fratti RA, McFalone M, Pagan-Ramos E, Deretic D, et al (1998) Effects of cytokines on mycobacterial phagosome maturation. J Cell Sci 111 (Pt 7): 897–905. 10.1242/jcs.111.7.897 9490634

[26] Mukherjee K, Parashuraman S, Krishnamurthy G, Majumdar J, Yadav A, et al (2002) Diverting intracellular trafficking of *Salmonella* to the lysosome through activation of the late endocytic Rab7 by intracellular delivery of muramyl dipeptide. J Cell Sci 115: 3693–3701. 12186955 10.1242/jcs.00034

[27] Desjardins M, Huber LA, Parton RG, Griffiths G (1994) Biogenesis of phagolysosomes proceeds through a sequential series of interactions with the endocytic apparatus. J Cell Biol 124: 677–688. 8120091 10.1083/jcb.124.5.677 PMC2119957

[28] Desjardins M, Nzala NN, Corsini R, Rondeau C (1997) Maturation of phagosomes is accompanied by changes in their fusion properties and size-selective acquisition of solute materials from endosomes. J Cell Sci 110 (Pt 18): 2303–2314. 10.1242/jcs.110.18.2303 9378779

[29] Desjardins M (1995) Biogenesis of phagolysosomes: the 'kiss and run' hypothesis. Trends Cell Biol 5: 183–186. 14731444 10.1016/s0962-8924(00)88989-8

[30] Seto S, Tsujimura K, Koide Y (2011) [Mechanism of intracellular parasitism by *Mycobacterium tuberculosis*]. Nihon Rinsho 69: 1373–1377. 21838032

[31] Deretic V, Fratti RA (1999) *Mycobacterium tuberculosis* phagosome. Mol Microbiol 31: 1603–1609. 10209735 10.1046/j.1365-2958.1999.01279.x

[32] Deretic V, Via LE, Fratti RA, Deretic D (1997) Mycobacterial phagosome maturation, rab proteins, and intracellular trafficking. Electrophoresis 18: 2542–2547. 9527483 10.1002/elps.1150181409

[33] Pethe K, Swenson DL, Alonso S, Anderson J, Wang C, et al (2004) Isolation of *Mycobacterium tuberculosis* mutants defective in the arrest of phagosome maturation. Proc Natl Acad Sci U S A 101: 13642–13647. 15340136 10.1073/pnas.0401657101 PMC518761

[34] Vergne I, Chua J, Deretic V (2003) *Mycobacterium tuberculosis* phagosome maturation arrest: selective targeting of PI3P-dependent membrane trafficking. Traffic 4: 600–606. 12911814 10.1034/j.1600-0854.2003.00120.x

[35] Indrigo J, Hunter RL Jr, Actor JK (2003) Cord factor trehalose 6,6′-dimycolate (TDM) mediates trafficking events during mycobacterial infection of murine macrophages. Microbiology 149: 2049–2059. 12904545 10.1099/mic.0.26226-0

[36] Walburger A, Koul A, Ferrari G, Nguyen L, Prescianotto-Baschong C, et al (2004) Protein kinase G from pathogenic mycobacteria promotes survival within macrophages. Science 304: 1800–1804. 15155913 10.1126/science.1099384

[37] Deghmane AE, Soualhine H, Bach H, Sendide K, Itoh S, et al (2007) Lipoamide dehydrogenase mediates retention of coronin-1 on BCG vacuoles, leading to arrest in phagosome maturation. J Cell Sci 120: 2796–2806. 17652161 10.1242/jcs.006221

[38] Master SS, Rampini SK, Davis AS, Keller C, Ehlers S, et al (2008) *Mycobacterium tuberculosis* prevents inflammasome activation. Cell Host Microbe 3: 224–232. 18407066 10.1016/j.chom.2008.03.003 PMC3657562

[39] Bach H, Papavinasasundaram KG, Wong D, Hmama Z, Av-Gay Y (2008) *Mycobacterium tuberculosis* virulence is mediated by PtpA dephosphorylation of human vacuolar protein sorting 33B. Cell Host Microbe 3: 316–322. 18474358 10.1016/j.chom.2008.03.008

[40] Tan T, Lee WL, Alexander DC, Grinstein S, Liu J (2006) The ESAT-6/CFP-10 secretion system of *Mycobacterium marinum* modulates phagosome maturation. Cell Microbiol 8: 1417–1429. 16922861 10.1111/j.1462-5822.2006.00721.x

[41] Palanisamy GS, Smith EE, Shanley CA, Ordway DJ, Orme IM, et al (2008) Disseminated disease severity as a measure of virulence of *Mycobacterium tuberculosis* in the guinea pig model. Tuberculosis (Edinb) 88: 295–306. 18321783 10.1016/j.tube.2007.12.003 PMC2572689

[42] McMurray DN (2001) Disease model: pulmonary tuberculosis. Trends Mol Med 7: 135–137. 11286786 10.1016/s1471-4914(00)01901-8

[43] Padilla-Carlin DJ, McMurray DN, Hickey AJ (2008) The guinea pig as a model of infectious diseases. Comp Med 58: 324–340. 18724774 PMC2706043

[44] Lewis KN, Liao R, Guinn KM, Hickey MJ, Smith S, et al (2003) Deletion of RD1 from *Mycobacterium tuberculosis* mimics bacille Calmette-Guerin attenuation. J Infect Dis 187: 117–123. 12508154 10.1086/345862 PMC1458498

[45] North RJ, Ryan L, LaCource R, Mogues T, Goodrich ME (1999) Growth rate of mycobacteria in mice as an unreliable indicator of mycobacterial virulence. Infect Immun 67: 5483–5485. 10496935 10.1128/iai.67.10.5483-5485.1999 PMC96910

[46] Warner DF, Mizrahi V (2006) Tuberculosis chemotherapy: the influence of bacillary stress and damage response pathways on drug efficacy. Clin Microbiol Rev 19: 558–570. 16847086 10.1128/CMR.00060-05 PMC1539104

